# Ethyl 7-oxo-3,5-diphenyl-1,4-diazepane-2-carboxyl­ate

**DOI:** 10.1107/S1600536812017084

**Published:** 2012-04-25

**Authors:** G. Jagadeesan, K. Sethusankar, P. Selvakumar, S. Thennarasu, A. B. Mandal

**Affiliations:** a Department of Physics, Dr MGR Educational and Research Institute, Dr MGR University, Chennai 600 095, India; b Department of Physics, RKM Vivekananda College (Autonomous), Chennai 600 004, India; c Organic Chemistry Division, Central Leather Research Institute, Adyar, Chennai 600 020, India

## Abstract

The title compound, C_20_H_22_N_2_O_3_, crystallizes with two independent mol­ecules in the asymmetric unit. In both mol­ecules, the diazepane rings adopt chair conformations. The mean planes of the diazepane rings in the two molecules form dihedral angles of 71.6 (4)/40.3 (5) and 75.9 (5)/58.6 (7)° with the neighbouring benzene rings. The carbonyl-group O atoms deviate significantly from the diazepane rings, by 0.685 (14) and 0.498 (13) Å. The eth­oxy­carbonyl groups show conformational difference between two mol­ecules, as reflected in the orientation of the carbonyl O atoms and the C—C—O—C torsion angle of −179.0 (2)° in one mol­ecule and 73.2 (2)° in the other. In one molecule there is a short N—H⋯O contact that generates an *S*(5) ring motif. In the crystal, N—H⋯O inter­actions generate *R*_2_^2^(8) graph-set motifs and C—H⋯O inter­actions generate *R*_2_^2^(10) and *R*_2_^2^(14) graph-set motifs. C—H⋯π inter­actions also occur.

## Related literature

For the biological importance of diazepanes, see: Wlodarczyk *et al.* (2005 ▶); Gopalakrishnan *et al.* (2007 ▶). For a related structure, see: Kumar *et al.* (2009 ▶). For puckering parameters, see: Cremer & Pople (1975 ▶). For graph-set notation, see: Bernstein *et al.* (1995 ▶).
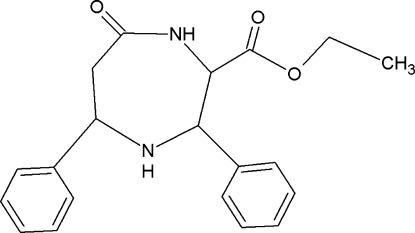


## Experimental

### 

#### Crystal data

C_20_H_22_N_2_O_3_
*M* *_r_* = 338.40
Triclinic, 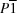

*a* = 9.5352 (3) Å
*b* = 14.8809 (4) Å
*c* = 15.0800 (4) Å
α = 61.650 (1)°
β = 82.153 (2)°
γ = 71.344 (2)°
*V* = 1783.86 (9) Å^3^
*Z* = 4
Mo *K*α radiation
μ = 0.09 mm^−1^
*T* = 293 K
0.30 × 0.30 × 0.25 mm

#### Data collection

Bruker Kappa APEXII CCD diffractometer
Absorption correction: multi-scan (*SADABS*; Bruker, 2008 ▶) *T*_min_ = 0.975, *T*_max_ = 0.979
39936 measured reflections
9437 independent reflections
6221 reflections with *I* > 2σ(*I*)
*R*_int_ = 0.032

#### Refinement

*R*[*F* ^2^ > 2σ(*F* ^2^)] = 0.052
*wR*(*F* ^2^) = 0.181
*S* = 1.15
9437 reflections
465 parameters
H atoms treated by a mixture of independent and constrained refinement
Δρ_max_ = 0.30 e Å^−3^
Δρ_min_ = −0.29 e Å^−3^

### 

Data collection: *APEX2* (Bruker, 2008 ▶); cell refinement: *SAINT* (Bruker, 2008 ▶); data reduction: *SAINT*; program(s) used to solve structure: *SHELXS97* (Sheldrick, 2008 ▶); program(s) used to refine structure: *SHELXL97* (Sheldrick, 2008 ▶); molecular graphics: *ORTEP-3* (Farrugia, 1997 ▶); software used to prepare material for publication: *SHELXL97* and *PLATON* (Spek, 2009 ▶).

## Supplementary Material

Crystal structure: contains datablock(s) global, I. DOI: 10.1107/S1600536812017084/pv2527sup1.cif

Structure factors: contains datablock(s) I. DOI: 10.1107/S1600536812017084/pv2527Isup2.hkl

Supplementary material file. DOI: 10.1107/S1600536812017084/pv2527Isup3.cml

Additional supplementary materials:  crystallographic information; 3D view; checkCIF report

## Figures and Tables

**Table 1:** Hydrogen-bond geometry (Å, °) *Cg*1 and *Cg*2 are the centroids of the C33–C38 and C26–C31 rings, respectively.

*D*—H⋯*A*	*D*—H	H⋯*A*	*D*⋯*A*	*D*—H⋯*A*
N2—H2⋯O2	0.85 (2)	2.45 (2)	2.776 (2)	104 (2)
N2—H2⋯O6^i^	0.85 (2)	2.26 (2)	3.091 (1)	170 (2)
N4—H4*A*⋯O3^ii^	0.85 (2)	2.14 (2)	2.983 (1)	169 (2)
C2—H2*B*⋯O2^iii^	0.97	2.53	3.233 (2)	130
C39—H39*A*⋯O5^iv^	0.97	2.48	3.355 (2)	150
C9—H9⋯*Cg*1^v^	0.93	2.89	3.735 (3)	152
C16—H16⋯*Cg*2^vi^	0.93	2.91	3.712 (1)	146

Symmetry codes: (i) 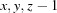; (ii) 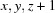; (iii) 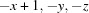; (iv) 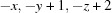; (v) 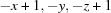; (vi) 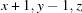.

**Fig. 1.**
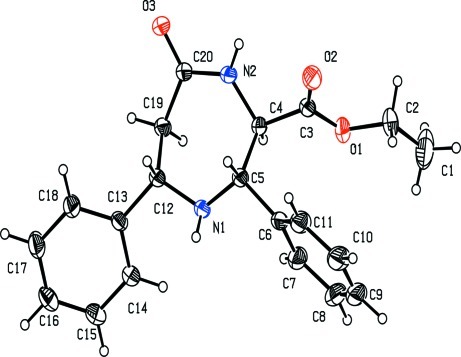
Molecule 1 of the title compound. Displacement ellipsoids are drawn at the 30% probability level.

**Fig. 2.**
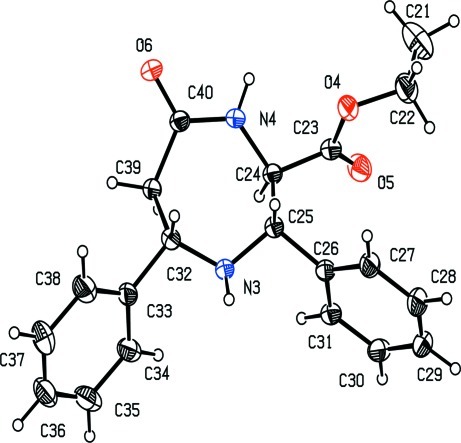
Molecule 2 of the title compound. Displacement ellipsoids are drawn at the 30% probability level.

**Fig. 3.**
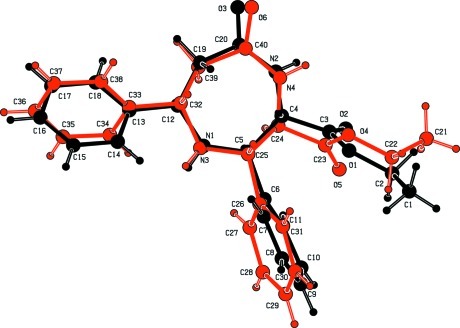
Molecule 1(Black) and Molecule 2 (Red) of the title compound overlapping each other, H atoms are shown as spheres of arbitrary radius.

**Fig. 4.**
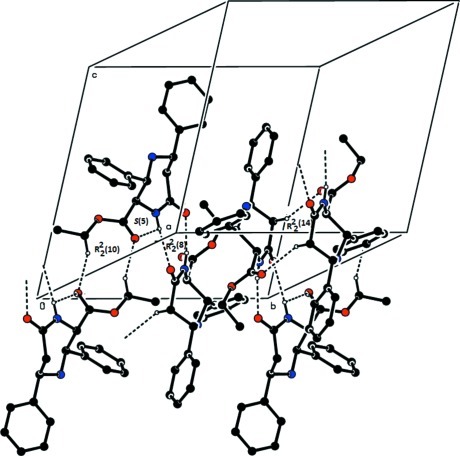
The crystal packing of the title compound viewed down the c axis, showing hydrogen bonds resulting in S(5) ring motif, dimers R22(8), R22(10) and R22(14) graphset motifs; H-atoms not involved in hydrogen bonds have been excluded for clarity.

## supplementary crystallographic information

### Comment

The title compound belongs to an important class of heterocyclic compounds that have widespread applications from pharmaceuticals (Wlodarczyk *et al.*, 2005) to biology (Gopalakrishnan *et al.*, 2007).

In the title structure there are two crystallographically independent molecules (1 and 2) in an asymmetric unit (Fig. 1 and Fig. 2, respectively). The central diazepane rings (C4/C5/C12/C19/C20/N1/N2) and (C24/C25/C32/C39/C40/N3/N4) in the molecule 1 and 2 form dihedral angles of 71.6 (4)°, 40.3 (5)° and 75.9 (5)°, 58.6 (7)° with the neighbouring benzene rings (C6–C11), (C13–C18) and (C26–C31), (C33–C38), respectively. The dihedral angles between the pairs of benzene rings (C6–C11), (C13–C18) and (C26–C31), (C33–C38) are 54.8 (7)° and 58.4 (6)°, respectively. The sum of the bond angles around the atoms N_2_ (362°) and N_4_ (359.8°) of the diazepane rings indicate *sp*^2^ hybridization, whereas the other N atoms, [N_1_ (328.6°) and N_3_ (331.2°)] indicate *sp*^3^ hybridization.

The atoms O3 and O6 deviate by 0.685 (14) Å and 0.498 (13) Å from the least square plane of the diazepane rings (C4/C5/C12/C19/C20/N1/N2) and (C24/C25/C32/C39/C40/N3/N4), respectively. The ethyl carboxylate groups exhibit significant conformational difference between the two molecules as reflected by the orientation of the carbonyl O-atoms and the torsion angles of C1—C2—O1—C3 is -179.0 (2)° for molecule 1 and C21—C22—O4—C23 is 73.2 (2)° in molecule 2. The conformational differences in the two molecules of the asymmetric unit of the title compound are evident in Fig. 3.

The diazepane rings (C4/C5/C12/C19/C20/N1/N2) and (C24/C25/C32/C39/C40/N3/N4) adopt *chair* conformations with puckering parameters (Cremer & Pople, 1975) *Q*_2_ = 0.382 (2) Å, *Q*_3_ = 0.678 (2) Å, φ_2_ = 180.4 (2)°, φ_3_ = 359.71 (14)° and *Q*_2_ = 0.333 (2) Å, *Q*_3_ = 0.670 (2) Å, φ_2_ = 182.9 (3)°, φ_3_ = 2.44 (14)°, respectively. The title compound exhibits structural similarities with another reported structure (Kumar *et al.*, 2009).

The crystal packing is stabilized by intramolecular N–H···O, intermolecular N–H···O, C–H···O and C–H···π interactions (Table 1). The N2–H2···O2, N2–H2···O6^i^, N2–H4A···O3^ii^, C2–H2B···O2^iii^ and C39–H39B···O5^iv^ hydrogen bonds generates S(5) ring motif, dimers *R*^2^_2_(8), *R*^2^_2_(10) and *R*^2^_2_(14) graphset motifs, respectively (Bernstein, *et al.*, 1995). The crystal packing is further stabilized by C9—H9···*Cg*1^v^ and C16—H16···*Cg*2^vi^ interactions where *Cg*1 is center of gravity of (C33–C38) ring and *Cg*2 is center of gravity of (C26–C31) ring (Fig. 4; symmetry codes are given in Table 1)

### Experimental

In a typical reaction, powdered ethyl 4-oxo-2,6-diphenyl piperidine- 3-carboxylate hydrochloride (2 g) was dissolved in an ice cold conc. H_2_SO_4_ (10 ml) in chloroform (5 ml) placed in a conical flask equipped with a magnetic stirrer. After the complete dissolution, the temperature of the solution was brought to 298 K. Sodium azide (600 mg) was added in portions over a period of 20 minutes with vigorous stirring. After the addition was over, the solution was poured slowly on to crushed ice with vigorous stirring, and the PH was adjusted to 8.0 using 4 N sodium hydroxide and extracted with chloroform. The combined organic layer was dried over sodium sulfate and evaporated to get the crude product which was purified by recrystallization from benzene and ethanol (1:1) to afford colourless prisms of the title compound.

### Refinement

The Hydrogen atoms were placed in calculated positions with C–H = 0.93 to 0.97 Å and N–H = 0.83 (2) to 0.85 (2) Å refined in the riding model with fixed isotropic displacement parameters: *U*_iso_(H) = 1.5 *U*_eq_(C) for methyl group and *U*_iso_(H) = 1.2 *U*_eq_(C/N) for other groups.

### Crystal data

**Table:**

C_20_H_22_N_2_O_3_	*Z* = 4
*M**_r_* = 338.40	*F*(000) = 720
Triclinic, *P*1	*D*_x_ = 1.260 Mg m^−^^3^
Hall symbol: -P 1	Mo *K*α radiation, λ = 0.71073 Å
*a* = 9.5352 (3) Å	Cell parameters from 9437 reflections
*b* = 14.8809 (4) Å	θ = 1.5–29.1°
*c* = 15.0800 (4) Å	µ = 0.09 mm^−^^1^
α = 61.650 (1)°	*T* = 293 K
β = 82.153 (2)°	Block, colourless
γ = 71.344 (2)°	0.30 × 0.30 × 0.25 mm
*V* = 1783.86 (9) Å^3^	

### Data collection

**Table:**

Bruker Kappa APEXII CCD diffractometer	9437 independent reflections
Radiation source: fine-focus sealed tube	6221 reflections with *I* > 2σ(*I*)
Graphite monochromator	*R*_int_ = 0.032
ω and φ scan	θ_max_ = 29.1°, θ_min_ = 2.3°
Absorption correction: multi-scan (*SADABS*; Bruker, 2008)	*h* = −12→12
*T*_min_ = 0.975, *T*_max_ = 0.979	*k* = −20→20
39936 measured reflections	*l* = −20→20

### Refinement

**Table:**

Refinement on *F*^2^	Primary atom site location: structure-invariant direct methods
Least-squares matrix: full	Secondary atom site location: difference Fourier map
*R*[*F*^2^ > 2σ(*F*^2^)] = 0.052	Hydrogen site location: inferred from neighbouring sites
*wR*(*F*^2^) = 0.181	H atoms treated by a mixture of independent and constrained refinement
*S* = 1.15	*w* = 1/[σ^2^(*F*_o_^2^) + (0.1*P*)^2^] where *P* = (*F*_o_^2^ + 2*F*_c_^2^)/3
9437 reflections	(Δ/σ)_max_ = 0.001
465 parameters	Δρ_max_ = 0.30 e Å^−^^3^
0 restraints	Δρ_min_ = −0.29 e Å^−^^3^

### Special details

**Table:**

Geometry. All e.s.d.'s (except the e.s.d. in the dihedral angle between two l.s. planes) are estimated using the full covariance matrix. The cell e.s.d.'s are taken into account individually in the estimation of e.s.d.'s in distances, angles and torsion angles; correlations between e.s.d.'s in cell parameters are only used when they are defined by crystal symmetry. An approximate (isotropic) treatment of cell e.s.d.'s is used for estimating e.s.d.'s involving l.s. planes.
Refinement. Refinement of *F*^2^ against ALL reflections. The weighted *R*-factor *wR* and goodness of fit *S* are based on *F*^2^, conventional *R*-factors *R* are based on *F*, with *F* set to zero for negative *F*^2^. The threshold expression of *F*^2^ > σ(*F*^2^) is used only for calculating *R*-factors(gt) *etc*. and is not relevant to the choice of reflections for refinement. *R*-factors based on *F*^2^ are statistically about twice as large as those based on *F*, and *R*- factors based on ALL data will be even larger.

### Fractional atomic coordinates and isotropic or equivalent isotropic displacement parameters (Å^2^)

**Table:**

	*x*	*y*	*z*	*U*_iso_*/*U*_eq_	
O1	0.47991 (14)	−0.06939 (10)	0.20798 (8)	0.0525 (3)	
O2	0.59115 (16)	0.04761 (10)	0.09211 (8)	0.0624 (4)	
O3	0.49513 (15)	0.29409 (9)	0.22928 (9)	0.0569 (3)	
N1	0.74551 (15)	−0.04002 (10)	0.39917 (9)	0.0407 (3)	
H1	0.833 (2)	−0.0769 (15)	0.4161 (13)	0.049*	
N2	0.53465 (15)	0.14336 (10)	0.21778 (9)	0.0409 (3)	
H2	0.5204 (19)	0.1802 (14)	0.1547 (14)	0.049*	
C1	0.3938 (4)	−0.1898 (2)	0.1853 (2)	0.1086 (10)	
H1A	0.4398	−0.2441	0.2491	0.163*	
H1B	0.3960	−0.2216	0.1425	0.163*	
H1C	0.2930	−0.1564	0.1954	0.163*	
C2	0.4739 (3)	−0.10988 (17)	0.13802 (15)	0.0668 (6)	
H2A	0.5734	−0.1417	0.1220	0.080*	
H2B	0.4237	−0.0519	0.0760	0.080*	
C3	0.54711 (18)	0.00514 (12)	0.17573 (11)	0.0422 (4)	
C4	0.56892 (16)	0.02807 (11)	0.25989 (10)	0.0365 (3)	
H4	0.5006	0.0025	0.3144	0.044*	
C5	0.73055 (17)	−0.03196 (11)	0.30013 (10)	0.0375 (3)	
H5	0.7964	0.0074	0.2531	0.045*	
C6	0.77658 (17)	−0.14315 (12)	0.31021 (11)	0.0416 (4)	
C7	0.7214 (2)	−0.22217 (14)	0.38552 (13)	0.0564 (5)	
H7	0.6597	−0.2083	0.4337	0.068*	
C8	0.7573 (3)	−0.32131 (15)	0.38941 (15)	0.0744 (6)	
H8	0.7205	−0.3741	0.4408	0.089*	
C9	0.8465 (3)	−0.34294 (16)	0.31860 (17)	0.0778 (7)	
H9	0.8687	−0.4097	0.3210	0.093*	
C10	0.9023 (2)	−0.26639 (17)	0.24476 (16)	0.0714 (6)	
H10	0.9636	−0.2811	0.1968	0.086*	
C11	0.8689 (2)	−0.16731 (15)	0.24034 (13)	0.0552 (5)	
H11	0.9088	−0.1159	0.1898	0.066*	
C12	0.73176 (16)	0.05855 (11)	0.40290 (10)	0.0364 (3)	
H12	0.7962	0.0961	0.3522	0.044*	
C13	0.78172 (16)	0.03114 (12)	0.50683 (10)	0.0373 (3)	
C14	0.79566 (18)	−0.06809 (13)	0.58854 (11)	0.0456 (4)	
H14	0.7732	−0.1204	0.5809	0.055*	
C15	0.8426 (2)	−0.09052 (15)	0.68171 (12)	0.0549 (5)	
H15	0.8518	−0.1578	0.7360	0.066*	
C16	0.8757 (2)	−0.01420 (16)	0.69434 (12)	0.0571 (5)	
H16	0.9076	−0.0295	0.7569	0.069*	
C17	0.8614 (2)	0.08413 (16)	0.61463 (13)	0.0594 (5)	
H17	0.8825	0.1364	0.6231	0.071*	
C18	0.8156 (2)	0.10706 (14)	0.52086 (12)	0.0516 (4)	
H18	0.8075	0.1743	0.4668	0.062*	
C19	0.57133 (17)	0.13165 (12)	0.38076 (10)	0.0397 (3)	
H19A	0.5049	0.0881	0.4144	0.048*	
H19B	0.5567	0.1803	0.4092	0.048*	
C20	0.53007 (16)	0.19611 (12)	0.26997 (10)	0.0386 (3)	
O4	0.31252 (12)	0.53774 (10)	1.14341 (8)	0.0493 (3)	
O5	0.07097 (13)	0.55078 (10)	1.15916 (8)	0.0552 (3)	
O6	0.46013 (13)	0.30348 (9)	0.99326 (8)	0.0525 (3)	
N3	0.16447 (14)	0.63277 (10)	0.83956 (9)	0.0405 (3)	
H3	0.1599 (19)	0.6944 (15)	0.7926 (14)	0.049*	
N4	0.32947 (14)	0.43294 (10)	1.03308 (9)	0.0411 (3)	
H4A	0.3836 (19)	0.3987 (14)	1.0864 (13)	0.049*	
C21	0.2862 (3)	0.4781 (2)	1.32355 (16)	0.0918 (8)	
H21A	0.3543	0.4117	1.3302	0.138*	
H21B	0.1867	0.4742	1.3285	0.138*	
H21C	0.3055	0.4917	1.3762	0.138*	
C22	0.3050 (2)	0.56515 (18)	1.22498 (14)	0.0647 (5)	
H22A	0.2227	0.6287	1.2121	0.078*	
H22B	0.3952	0.5813	1.2267	0.078*	
C23	0.18582 (17)	0.53656 (12)	1.11706 (11)	0.0395 (3)	
C24	0.20218 (16)	0.52322 (11)	1.02292 (10)	0.0355 (3)	
H24	0.1137	0.5073	1.0154	0.043*	
C25	0.20763 (16)	0.63003 (11)	0.93032 (10)	0.0356 (3)	
H25	0.3083	0.6362	0.9232	0.043*	
C26	0.10097 (16)	0.72316 (11)	0.94280 (10)	0.0365 (3)	
C27	0.15310 (19)	0.78986 (13)	0.96197 (11)	0.0451 (4)	
H27	0.2544	0.7797	0.9635	0.054*	
C28	0.0547 (2)	0.87075 (14)	0.97860 (13)	0.0557 (5)	
H28	0.0902	0.9156	0.9903	0.067*	
C29	−0.0951 (2)	0.88640 (14)	0.97817 (13)	0.0571 (5)	
H29	−0.1602	0.9409	0.9904	0.069*	
C30	−0.14881 (19)	0.82081 (14)	0.95948 (12)	0.0506 (4)	
H30	−0.2501	0.8307	0.9591	0.061*	
C31	−0.05059 (17)	0.74068 (12)	0.94139 (11)	0.0421 (4)	
H31	−0.0869	0.6973	0.9279	0.050*	
C32	0.26852 (17)	0.56031 (12)	0.80412 (10)	0.0378 (3)	
H32	0.3678	0.5678	0.8009	0.045*	
C33	0.21848 (18)	0.59202 (12)	0.69885 (10)	0.0417 (4)	
C34	0.0713 (2)	0.63035 (13)	0.67137 (12)	0.0505 (4)	
H34	−0.0005	0.6397	0.7171	0.061*	
C35	0.0286 (2)	0.65530 (15)	0.57568 (14)	0.0651 (6)	
H35	−0.0714	0.6814	0.5582	0.078*	
C36	0.1311 (3)	0.64200 (16)	0.50766 (15)	0.0734 (6)	
H36	0.1019	0.6588	0.4439	0.088*	
C37	0.2760 (3)	0.6040 (2)	0.53387 (15)	0.0824 (7)	
H37	0.3472	0.5945	0.4879	0.099*	
C38	0.3196 (2)	0.57900 (18)	0.62977 (14)	0.0691 (6)	
H38	0.4199	0.5529	0.6467	0.083*	
C39	0.27301 (17)	0.44403 (11)	0.87378 (10)	0.0390 (3)	
H39A	0.1720	0.4414	0.8905	0.047*	
H39B	0.3131	0.4036	0.8365	0.047*	
C40	0.36168 (16)	0.38832 (11)	0.97132 (11)	0.0378 (3)	

### Atomic displacement parameters (Å^2^)

**Table:**

	*U*^11^	*U*^22^	*U*^33^	*U*^12^	*U*^13^	*U*^23^
O1	0.0665 (8)	0.0548 (7)	0.0509 (6)	−0.0214 (6)	−0.0054 (5)	−0.0318 (6)
O2	0.1019 (10)	0.0526 (8)	0.0345 (6)	−0.0243 (7)	−0.0070 (6)	−0.0182 (5)
O3	0.0821 (9)	0.0307 (6)	0.0520 (6)	−0.0023 (6)	−0.0264 (6)	−0.0162 (5)
N1	0.0499 (8)	0.0327 (7)	0.0367 (6)	0.0007 (6)	−0.0144 (5)	−0.0178 (5)
N2	0.0556 (8)	0.0297 (6)	0.0313 (6)	−0.0030 (6)	−0.0119 (5)	−0.0119 (5)
C1	0.164 (3)	0.109 (2)	0.107 (2)	−0.079 (2)	0.0090 (19)	−0.0683 (18)
C2	0.0872 (14)	0.0718 (13)	0.0651 (11)	−0.0244 (11)	−0.0112 (10)	−0.0460 (11)
C3	0.0520 (9)	0.0344 (8)	0.0383 (8)	−0.0021 (7)	−0.0133 (7)	−0.0182 (6)
C4	0.0466 (8)	0.0300 (7)	0.0321 (6)	−0.0068 (6)	−0.0058 (6)	−0.0148 (6)
C5	0.0448 (8)	0.0333 (8)	0.0322 (6)	−0.0049 (6)	−0.0059 (6)	−0.0158 (6)
C6	0.0503 (9)	0.0333 (8)	0.0366 (7)	0.0014 (7)	−0.0124 (6)	−0.0177 (6)
C7	0.0824 (13)	0.0420 (10)	0.0433 (8)	−0.0139 (9)	−0.0050 (8)	−0.0195 (8)
C8	0.1198 (19)	0.0369 (10)	0.0579 (11)	−0.0176 (11)	−0.0180 (12)	−0.0135 (9)
C9	0.1173 (19)	0.0424 (11)	0.0682 (13)	0.0069 (12)	−0.0304 (13)	−0.0318 (11)
C10	0.0825 (14)	0.0554 (13)	0.0681 (12)	0.0154 (11)	−0.0139 (11)	−0.0404 (11)
C11	0.0606 (11)	0.0472 (10)	0.0508 (9)	0.0030 (8)	−0.0065 (8)	−0.0270 (8)
C12	0.0437 (8)	0.0336 (8)	0.0296 (6)	−0.0073 (6)	−0.0062 (6)	−0.0133 (6)
C13	0.0389 (8)	0.0399 (8)	0.0318 (6)	−0.0070 (6)	−0.0048 (5)	−0.0169 (6)
C14	0.0535 (9)	0.0424 (9)	0.0381 (7)	−0.0110 (7)	−0.0083 (7)	−0.0159 (7)
C15	0.0673 (11)	0.0535 (11)	0.0351 (7)	−0.0152 (9)	−0.0103 (7)	−0.0121 (7)
C16	0.0647 (11)	0.0714 (12)	0.0373 (8)	−0.0169 (9)	−0.0089 (7)	−0.0259 (8)
C17	0.0796 (13)	0.0655 (12)	0.0489 (9)	−0.0297 (10)	−0.0047 (9)	−0.0319 (9)
C18	0.0729 (11)	0.0462 (10)	0.0401 (8)	−0.0219 (9)	−0.0068 (7)	−0.0185 (7)
C19	0.0473 (8)	0.0349 (8)	0.0345 (7)	−0.0031 (6)	−0.0063 (6)	−0.0178 (6)
C20	0.0433 (8)	0.0310 (8)	0.0377 (7)	−0.0040 (6)	−0.0089 (6)	−0.0147 (6)
O4	0.0457 (6)	0.0578 (7)	0.0484 (6)	−0.0068 (5)	−0.0069 (5)	−0.0307 (5)
O5	0.0463 (7)	0.0703 (9)	0.0469 (6)	−0.0138 (6)	0.0031 (5)	−0.0278 (6)
O6	0.0574 (7)	0.0369 (6)	0.0519 (6)	0.0084 (5)	−0.0180 (5)	−0.0208 (5)
N3	0.0532 (8)	0.0281 (6)	0.0330 (6)	−0.0011 (6)	−0.0095 (5)	−0.0127 (5)
N4	0.0457 (7)	0.0334 (7)	0.0376 (6)	0.0016 (5)	−0.0151 (5)	−0.0153 (5)
C21	0.0918 (17)	0.138 (2)	0.0565 (12)	−0.0486 (17)	−0.0013 (11)	−0.0430 (14)
C22	0.0572 (11)	0.0839 (15)	0.0658 (11)	−0.0121 (10)	−0.0084 (9)	−0.0473 (11)
C23	0.0407 (8)	0.0339 (8)	0.0371 (7)	−0.0056 (6)	−0.0057 (6)	−0.0125 (6)
C24	0.0371 (7)	0.0298 (7)	0.0365 (7)	−0.0056 (6)	−0.0067 (6)	−0.0135 (6)
C25	0.0392 (8)	0.0317 (7)	0.0345 (7)	−0.0078 (6)	−0.0035 (6)	−0.0147 (6)
C26	0.0438 (8)	0.0291 (7)	0.0308 (6)	−0.0078 (6)	−0.0028 (6)	−0.0102 (5)
C27	0.0544 (9)	0.0379 (9)	0.0448 (8)	−0.0139 (7)	−0.0019 (7)	−0.0195 (7)
C28	0.0743 (12)	0.0413 (10)	0.0583 (10)	−0.0139 (9)	−0.0057 (9)	−0.0283 (8)
C29	0.0729 (13)	0.0411 (10)	0.0493 (9)	0.0018 (9)	−0.0049 (8)	−0.0246 (8)
C30	0.0484 (9)	0.0466 (10)	0.0437 (8)	−0.0005 (8)	−0.0059 (7)	−0.0172 (7)
C31	0.0480 (9)	0.0337 (8)	0.0390 (7)	−0.0067 (7)	−0.0072 (6)	−0.0136 (6)
C32	0.0421 (8)	0.0361 (8)	0.0343 (7)	−0.0079 (6)	−0.0061 (6)	−0.0159 (6)
C33	0.0562 (9)	0.0323 (8)	0.0337 (7)	−0.0094 (7)	−0.0066 (6)	−0.0132 (6)
C34	0.0594 (10)	0.0439 (9)	0.0428 (8)	−0.0152 (8)	−0.0127 (7)	−0.0122 (7)
C35	0.0842 (14)	0.0527 (11)	0.0542 (10)	−0.0224 (10)	−0.0298 (10)	−0.0119 (9)
C36	0.1207 (19)	0.0562 (12)	0.0440 (9)	−0.0227 (12)	−0.0246 (11)	−0.0188 (9)
C37	0.1040 (18)	0.0973 (18)	0.0479 (10)	−0.0151 (14)	−0.0004 (11)	−0.0434 (11)
C38	0.0660 (12)	0.0847 (15)	0.0489 (10)	−0.0016 (10)	−0.0067 (9)	−0.0354 (10)
C39	0.0456 (8)	0.0322 (8)	0.0383 (7)	−0.0038 (6)	−0.0110 (6)	−0.0172 (6)
C40	0.0409 (8)	0.0299 (7)	0.0386 (7)	−0.0064 (6)	−0.0070 (6)	−0.0131 (6)

### Geometric parameters (Å, º)

**Table:**

O1—C3	1.317 (2)	O4—C23	1.3300 (18)
O1—C2	1.4566 (19)	O4—C22	1.456 (2)
O2—C3	1.1976 (19)	O5—C23	1.1986 (19)
O3—C20	1.2283 (18)	O6—C40	1.2306 (17)
N1—C12	1.4578 (19)	N3—C25	1.4609 (17)
N1—C5	1.4637 (16)	N3—C32	1.467 (2)
N1—H1	0.83 (2)	N3—H3	0.84 (2)
N2—C20	1.3389 (19)	N4—C40	1.3347 (19)
N2—C4	1.4550 (18)	N4—C24	1.4538 (18)
N2—H2	0.85 (2)	N4—H4A	0.85 (2)
C1—C2	1.461 (3)	C21—C22	1.472 (3)
C1—H1A	0.9600	C21—H21A	0.9600
C1—H1B	0.9600	C21—H21B	0.9600
C1—H1C	0.9600	C21—H21C	0.9600
C2—H2A	0.9700	C22—H22A	0.9700
C2—H2B	0.9700	C22—H22B	0.9700
C3—C4	1.5163 (19)	C23—C24	1.506 (2)
C4—C5	1.5496 (19)	C24—C25	1.551 (2)
C4—H4	0.9800	C24—H24	0.9800
C5—C6	1.506 (2)	C25—C26	1.511 (2)
C5—H5	0.9800	C25—H25	0.9800
C6—C7	1.384 (2)	C26—C31	1.385 (2)
C6—C11	1.385 (2)	C26—C27	1.394 (2)
C7—C8	1.377 (3)	C27—C28	1.376 (2)
C7—H7	0.9300	C27—H27	0.9300
C8—C9	1.368 (3)	C28—C29	1.372 (3)
C8—H8	0.9300	C28—H28	0.9300
C9—C10	1.357 (3)	C29—C30	1.384 (3)
C9—H9	0.9300	C29—H29	0.9300
C10—C11	1.375 (3)	C30—C31	1.378 (2)
C10—H10	0.9300	C30—H30	0.9300
C11—H11	0.9300	C31—H31	0.9300
C12—C13	1.5239 (17)	C32—C33	1.5236 (18)
C12—C19	1.538 (2)	C32—C39	1.529 (2)
C12—H12	0.9800	C32—H32	0.9800
C13—C18	1.380 (2)	C33—C38	1.360 (3)
C13—C14	1.381 (2)	C33—C34	1.375 (2)
C14—C15	1.3845 (19)	C34—C35	1.393 (2)
C14—H14	0.9300	C34—H34	0.9300
C15—C16	1.370 (3)	C35—C36	1.357 (3)
C15—H15	0.9300	C35—H35	0.9300
C16—C17	1.361 (3)	C36—C37	1.350 (3)
C16—H16	0.9300	C36—H36	0.9300
C17—C18	1.386 (2)	C37—C38	1.399 (2)
C17—H17	0.9300	C37—H37	0.9300
C18—H18	0.9300	C38—H38	0.9300
C19—C20	1.5107 (18)	C39—C40	1.5162 (17)
C19—H19A	0.9700	C39—H39A	0.9700
C19—H19B	0.9700	C39—H39B	0.9700
			
C3—O1—C2	115.98 (14)	C23—O4—C22	116.14 (13)
C12—N1—C5	117.12 (11)	C25—N3—C32	116.90 (11)
C12—N1—H1	105.4 (12)	C25—N3—H3	106.1 (12)
C5—N1—H1	103.2 (12)	C32—N3—H3	104.6 (12)
C20—N2—C4	125.40 (12)	C40—N4—C24	125.62 (11)
C20—N2—H2	116.9 (12)	C40—N4—H4A	114.4 (12)
C4—N2—H2	117.7 (12)	C24—N4—H4A	119.4 (12)
C2—C1—H1A	109.5	C22—C21—H21A	109.5
C2—C1—H1B	109.5	C22—C21—H21B	109.5
H1A—C1—H1B	109.5	H21A—C21—H21B	109.5
C2—C1—H1C	109.5	C22—C21—H21C	109.5
H1A—C1—H1C	109.5	H21A—C21—H21C	109.5
H1B—C1—H1C	109.5	H21B—C21—H21C	109.5
O1—C2—C1	108.45 (18)	O4—C22—C21	112.10 (18)
O1—C2—H2A	110.0	O4—C22—H22A	109.2
C1—C2—H2A	110.0	C21—C22—H22A	109.2
O1—C2—H2B	110.0	O4—C22—H22B	109.2
C1—C2—H2B	110.0	C21—C22—H22B	109.2
H2A—C2—H2B	108.4	H22A—C22—H22B	107.9
O2—C3—O1	125.40 (14)	O5—C23—O4	124.41 (14)
O2—C3—C4	123.26 (15)	O5—C23—C24	124.07 (13)
O1—C3—C4	111.28 (13)	O4—C23—C24	111.37 (13)
N2—C4—C3	107.04 (11)	N4—C24—C23	110.14 (11)
N2—C4—C5	112.95 (12)	N4—C24—C25	113.96 (12)
C3—C4—C5	108.33 (12)	C23—C24—C25	109.29 (12)
N2—C4—H4	109.5	N4—C24—H24	107.7
C3—C4—H4	109.5	C23—C24—H24	107.7
C5—C4—H4	109.5	C25—C24—H24	107.7
N1—C5—C6	108.35 (11)	N3—C25—C26	108.37 (11)
N1—C5—C4	110.42 (12)	N3—C25—C24	109.46 (11)
C6—C5—C4	110.90 (12)	C26—C25—C24	110.26 (12)
N1—C5—H5	109.0	N3—C25—H25	109.6
C6—C5—H5	109.0	C26—C25—H25	109.6
C4—C5—H5	109.0	C24—C25—H25	109.6
C7—C6—C11	118.05 (16)	C31—C26—C27	118.31 (14)
C7—C6—C5	121.24 (15)	C31—C26—C25	120.95 (13)
C11—C6—C5	120.60 (15)	C27—C26—C25	120.63 (13)
C8—C7—C6	120.29 (19)	C28—C27—C26	119.95 (16)
C8—C7—H7	119.9	C28—C27—H27	120.0
C6—C7—H7	119.9	C26—C27—H27	120.0
C9—C8—C7	120.7 (2)	C29—C28—C27	121.13 (17)
C9—C8—H8	119.6	C29—C28—H28	119.4
C7—C8—H8	119.6	C27—C28—H28	119.4
C10—C9—C8	119.57 (19)	C28—C29—C30	119.71 (16)
C10—C9—H9	120.2	C28—C29—H29	120.1
C8—C9—H9	120.2	C30—C29—H29	120.1
C9—C10—C11	120.5 (2)	C31—C30—C29	119.30 (16)
C9—C10—H10	119.8	C31—C30—H30	120.4
C11—C10—H10	119.8	C29—C30—H30	120.4
C10—C11—C6	120.86 (19)	C30—C31—C26	121.60 (15)
C10—C11—H11	119.6	C30—C31—H31	119.2
C6—C11—H11	119.6	C26—C31—H31	119.2
N1—C12—C13	109.03 (11)	N3—C32—C33	108.08 (11)
N1—C12—C19	111.03 (12)	N3—C32—C39	111.54 (13)
C13—C12—C19	110.09 (11)	C33—C32—C39	109.35 (11)
N1—C12—H12	108.9	N3—C32—H32	109.3
C13—C12—H12	108.9	C33—C32—H32	109.3
C19—C12—H12	108.9	C39—C32—H32	109.3
C18—C13—C14	118.12 (13)	C38—C33—C34	117.67 (14)
C18—C13—C12	119.56 (13)	C38—C33—C32	120.40 (14)
C14—C13—C12	122.33 (13)	C34—C33—C32	121.88 (14)
C13—C14—C15	120.77 (15)	C33—C34—C35	120.62 (18)
C13—C14—H14	119.6	C33—C34—H34	119.7
C15—C14—H14	119.6	C35—C34—H34	119.7
C16—C15—C14	120.35 (16)	C36—C35—C34	120.89 (19)
C16—C15—H15	119.8	C36—C35—H35	119.6
C14—C15—H15	119.8	C34—C35—H35	119.6
C17—C16—C15	119.46 (15)	C37—C36—C35	119.06 (17)
C17—C16—H16	120.3	C37—C36—H36	120.5
C15—C16—H16	120.3	C35—C36—H36	120.5
C16—C17—C18	120.59 (16)	C36—C37—C38	120.3 (2)
C16—C17—H17	119.7	C36—C37—H37	119.8
C18—C17—H17	119.7	C38—C37—H37	119.8
C13—C18—C17	120.70 (15)	C33—C38—C37	121.42 (19)
C13—C18—H18	119.7	C33—C38—H38	119.3
C17—C18—H18	119.7	C37—C38—H38	119.3
C20—C19—C12	114.10 (12)	C40—C39—C32	116.77 (12)
C20—C19—H19A	108.7	C40—C39—H39A	108.1
C12—C19—H19A	108.7	C32—C39—H39A	108.1
C20—C19—H19B	108.7	C40—C39—H39B	108.1
C12—C19—H19B	108.7	C32—C39—H39B	108.1
H19A—C19—H19B	107.6	H39A—C39—H39B	107.3
O3—C20—N2	121.37 (13)	O6—C40—N4	120.90 (12)
O3—C20—C19	120.51 (13)	O6—C40—C39	120.65 (13)
N2—C20—C19	118.12 (13)	N4—C40—C39	118.44 (13)

### Hydrogen-bond geometry (Å, º)

Cg1 and Cg2 are the centroids of the C33–C38 and C26–C31 rings, respectively.

**Table:**

*D*—H···*A*	*D*—H	H···*A*	*D*···*A*	*D*—H···*A*
N2—H2···O2	0.85 (2)	2.45 (2)	2.776 (2)	104
N2—H2···O6^i^	0.85 (2)	2.26 (2)	3.091 (1)	170
N4—H4*A*···O3^ii^	0.85 (2)	2.14 (2)	2.983 (1)	169
C2—H2*B*···O2^iii^	0.97	2.53	3.233 (2)	130
C39—H39*A*···O5^iv^	0.97	2.48	3.355 (2)	150
C9—H9···*Cg*1^v^	0.93	2.89	3.735 (3)	152
C16—H16···*Cg*2^vi^	0.93	2.91	3.712 (1)	146

Symmetry codes: (i) *x*, *y*, *z*−1; (ii) *x*, *y*, *z*+1; (iii) −*x*+1, −*y*, −*z*; (iv) −*x*, −*y*+1, −*z*+2; (v) −*x*+1, −*y*, −*z*+1; (vi) *x*+1, *y*−1, *z*.
